# Porous α-Fe_2_O_3_@C Nanowire Arrays as Flexible Supercapacitors Electrode Materials with Excellent Electrochemical Performances

**DOI:** 10.3390/nano8070487

**Published:** 2018-07-01

**Authors:** Yidi Dong, Lei Xing, Kunfeng Chen, Xiang Wu

**Affiliations:** 1 School of Materials Science and Engineering, Shenyang University of Technology, Shenyang 110870, China; dyd19941227@163.com (Y.D.); xinglei195914@163.com (L.X.); 2 State Key Laboratory of Rare Earth Resource Utilization, Changchun Institute of Applied Chemistry, Chinese Academy of Science, Changchun 130022, China; kfchen@ciac.ac.cn

**Keywords:** α-Fe_2_O_3_/C, asymmetrical supercapacitor, long cycling life, energy storage device

## Abstract

Porous α-Fe_2_O_3_ nanowire arrays coated with a layer of carbon shell have been prepared by a simple hydrothermal route. The as-synthesized products show an excellent electrochemical performance with high specific capacitance and good cycling life after 9000 cycles. A solid state asymmetric supercapacitor (ASC) with a 2 V operation voltage window has been assembled by porous α-Fe_2_O_3_/C nanowire arrays as the anode materials, and MnO_2_ nanosheets as the cathode materials, which gives rise to a maximum energy density of 30.625 Wh kg^−1^and a maximum power density of 5000 W kg^−1^ with an excellent cycling performance of 82% retention after 10,000 cycles.

## 1. Introduction

Supercapacitors (SCs) as a promising energy storage device with advantages of fast charge-discharge rate, long cycle life and high-power density, which can be applied in various portable energy source supplies [1,2,3,4,5,6]. Asymmetric supercapacitors (ASCs) have attracted widespread attention due to their potential applications in hybrid electric vehicles, microelectromechamical systems, sensors and flexible electronics [7,8,9,10]. In general, ASCs consist of a battery-type Faradaic cathode as an energy source and a double layer type anode as the power source, which can be operated in a wide working voltage window and delivers a high level of energy density [11,12,13,14]. Therefore, the exploration of high performance anode and cathode materials has attracted considerable interest. The mesoporous oxide nanomaterials with a high specific surface area can increase charge accumulation and ion transmission rates, which can be widely applied in ASCs [15,16]. To date, many efforts have been made to design a variety of layered nano/microarchitectures for ASCs electrodes. Among them, metal oxides might provide a variety of redox reactions for supercapacitors [17,18,19]. However, supercapacitor devices still have a low energy density, which limits the popularization of supercapacitors. The limited reports and poor performance indicate that there is an urgent need to further explore ASCs’ high-performance negative electrodes.

Hematite (α-Fe_2_O_3_) is an environmentally friendly, low cost, non-toxic and stable electrode material. It possesses a high theoretical capacitance (3265 F g^−1^) and a suitable negative potential working voltage (−1.0 V–0 V) [20,21,22,23,24]. At present, α-Fe_2_O_3_ has received extensive attention as high performance anode material for ASCs. However, α-Fe_2_O_3_ has a lower conductivity, which makes the reported α-Fe_2_O_3_ electrode material possess a lower capacitance. Therefore, some strategies have been taken to improve the capacitance of α-Fe_2_O_3_ materials, including designing the microstructure of the materials to shorten the transmission path of ion electrons; coating the surface with conductive polymers or carbon materials to improve the conductivity of the materials; and oxygen vacancies being introduced or doped with other metal elements [25,26,27,28,29]. However, the specific capacitance obtained is still far below the theoretical capacitance. Therefore, improving the capacity of α-Fe_2_O_3_ electrode materials while still maintaining its capacity remains a huge challenge.

In this work, we report a carbon-coated α-Fe_2_O_3_ (α-Fe_2_O_3_@C) core/shell nanowire arrays grown on a flexible carbon cloth by using a simple hydrothermal method. The conductivity of α-Fe_2_O_3_ can be significantly improved by coating a carbon shell. Therefore, its capacitance performance can be improved. The as-prepared α-Fe_2_O_3_@C nanostructures show a high capacitance of 280 F g^−1^ at a current density of 1 A g^−1^, and ASCs are assembled, which show the maximum energy density of 30.625 Wh kg^−1^ and the maximum power density of 5000 W kg^−1^.

## 2. Experimental Details

### 2.1. Preparation of α-Fe_2_O_3_ Samples

α-Fe_2_O_3_ nanowire arrays were synthesized by a hydrothermal method. Typically, 1.7675 g of Fe(NO_3_)_3_·9H_2_O and 0.6214 g Na_2_SO_4_ were dissolved in 80 mL of deionized water with magnetic stirring for 20 min. Then, the as-obtained yellow solution was transferred into a 100 mL Teflon-lined stainless-steel autoclave. A piece of clean carbon cloth (2 cm × 3 cm) was dipped into the above-mentioned solution in the autoclave and kept at 120 °C for 6 h. After cooling to room temperature naturally, the samples were repeatedly rinsed with deionized water and dried at 60 °C in air. The final products were annealed at 300 °C for 2 h in air.

### 2.2. Preparation of α-Fe_2_O_3_@C Composite

First, 0.5 g glucose was dispersed in 80 mL of deionized water with magnetic stirring for 10 min. The above-mentioned solution was transferred into a 100 mL Teflon-lined stainless steel autoclave. Then, the carbon cloth with α-Fe_2_O_3_ nanowire arrays was put into the autoclave for hydrothermal treatment at180 °C for 4 h. Final products were obtained by centrifugation and several washes with deionized water and alcohol, respectively, and then dried at 80 °C for 12 h. After that, the as-synthesized sample was further sintered at 450 °C in an Ar atmosphere for 2 h.

### 2.3. Preparation of MnO_2_ Nanosheets

0.236 g KMnO_4_ was dispersed in 80 mL of deionized water with magnetic stirring for 20 min. A piece of clean carbon cloth (2 cm × 3 cm) was dipped into the above-mentioned solution. Then, the as-obtained solution was transferred into a 100 mL Teflon-lined autoclave for hydrothermal treatment at 180 °C for 5 h. After cooling to room temperature, the carbon cloth was washed with deionized water several times, and dried at 80 °C. The final products were annealed at 300 °C for 2 h in air.

### 2.4. Characterizations

The morphology and microstructure of the products were observed using scanning electron microscope (SEM; Hitachi, Japan, SU8010). The crystal structure and phase purity of the samples were characterized by X-ray diffraction (XRD) with Cu Kα (λ = 1.5478 Å). An electrochemical work station (CHI660E, Shanghai Chenhua, China) was used to test the electrochemical properties of the as-prepared electrode materials.

### 2.5. Electrochemical Measurement

For the three electrode system tests, porous α-Fe_2_O_3_@C nanowire arrays were directly used as the working electrode, Pt foil was used as the counter electrode, and Ag/AgCl as the reference electrode, respectively. The electrode area is 1 cm^2^. Electrochemical measurements were tested on a electrochemical workstation in a 1M Na_2_SO_4_ aqueous solution, and all of the experiments were tested at room temperature. Electrochemical behaviors of the as-obtained samples were investigated by cycling voltammetry (CV) and galvanostatic charge-discharge (GCD) measurement. The applied potential window was ranged from −1 to 0 V. CV curves were presented at scan rates of 5, 10, 20, 50 and 100 mVs^−1^. GCD curves were measured at current densities of 1, 2, 3, 4 and 5 Ag^−1^. Electrochemical impedance spectroscopy (EIS) was measured between 10^2^ KHz and 10^−5^ KHz with an amplitude of 10 mV. Specific capacitance of the electrode can be calculated from galvanostatic charge-discharge curves based on the following equation:

(1) $$C_{\mathrm{SP}}=\frac{I\Delta t}{\Delta m\Delta V}$$

(2) $$C_{a}=\frac{I\Delta t}{\Delta S\Delta V}$$

where *I*, Δ*t*, Δ*m* Δ*S* and Δ*V* refer to applied current (A), discharged time (s), the mass of active materials, the area of the working electrode and sweep potential window, in addition, *C*_sp_ is the mass specific capacitance, and *C*_a_ is the area capacitance.

### 2.6. Assembly of α-Fe_2_O_3_@C Nanowire Arrays//MnO_2_ Nanosheets ASCs

The as-fabricated quasi-solid state ASCs were assembled using MnO_2_nanosheets as cathode electrode materials, α-Fe_2_O_3_@C nanowire arrays as anode electrode materials and the separator (NKK, Nippon Kodoshi Corporation, Kochi, Japan). The PVA/Na_2_SO_4_ electrolyte gel was fabricated by adding Na_2_SO_4_ (2.13 g) and PVA (3 g) into deionized water (50 mL), respectively and heating at 90°C for 2 h under constant stirring. The mass loading of the α-Fe_2_O_3_@C and MnO_2_ was determined based on the charge balance principle, as shown in Formulas (3) and (4) [30,31]:

(3) $$q^{+}=q^{-}$$

(4) $$q^{+}=C\times \Delta V\times m_{+}$$

*q*, *C*, Δ*V* and *m* are the amount of the stored charge (*C*), the specific capacitance of the electrode (F g^−1^), the working voltage window (*V*), the mass loading of the active materials (g), respectively.

And corresponding energy density *E* (Wh/kg) and power density *P* (W/kg) are calculated from the equations:

(5) $$E=\frac{CV^{2}}{2}$$

(6) $$P=\frac{E}{\Delta t}$$

*C* stands for specific capacitance, *V* is potential change and Δ*t* is discharge time.

## 3. Results and Discussion

**Figure 1:**
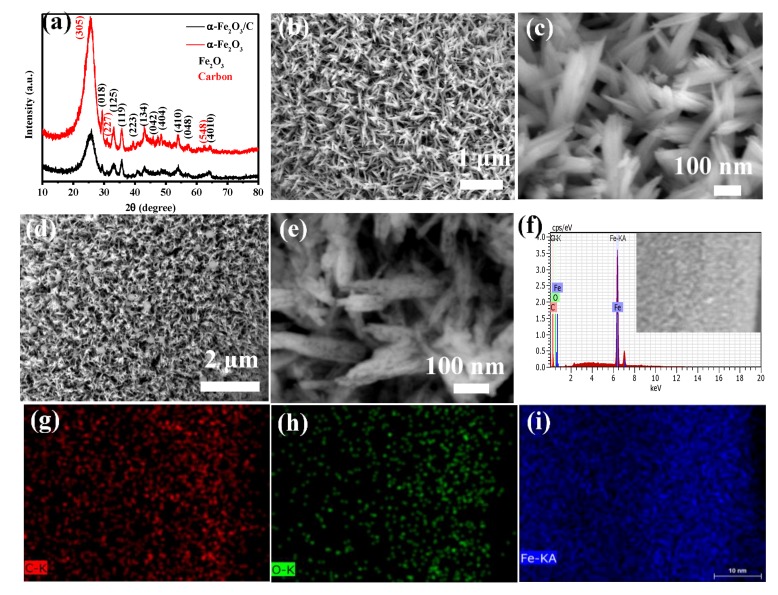
(**a**) XRD patterns of α-Fe_2_O_3_ and α-Fe_2_O_3_@C nanowire arrays (**b**,**c**) SEM image of α-Fe_2_O_3_ nanowire arrays (**d**,**e**) SEM images ofα-Fe_2_O_3_@C nanowire arrays (**f**–**i**) EDS mapping of α-Fe_2_O_3_ nanowire arrays.

The phase and composition of the as-synthesized products are confirmed by XRD. In Figure 1a, the characteristic peaks of hexagonal α-Fe_2_O_3_ phase (PDF card no. 33-0664) and characteristic peaks of C phase (PDF card no. 22-1069) are clearly seen in the XRD spectra. From the XRD spectra of α-Fe_2_O_3_@C products, it can be seen that the peaks position of XRD spectrum does not change after carbon coating, but the shape of the peaks become sharper, which is because after the second-high temperature calcination, the crystallinity of the products improves. The SEM image of α-Fe_2_O_3_ nanowire arrays is shown in Figure 1b,c, where it can be seen that α-Fe_2_O_3_ nanowire with an average diameter of 80 nm and the length of 300 nm are grown on the surface of carbon cloth. Figure 1d,e shows SEM image of α-Fe_2_O_3_@C products, it can be seen that after carbon coating, the surface of nanowire arrays become rougher. And there are more pores on the surface of the nanowires, which can provide more paths for electron transmission. To confirm the element composition of the as-synthesized sample, EDS element mapping is conducted. From the EDS spectra of the sample (Figure 1f–i), the composition of C, O and Fe elements are evidently presented and well-distributed.

**Figure 2:**
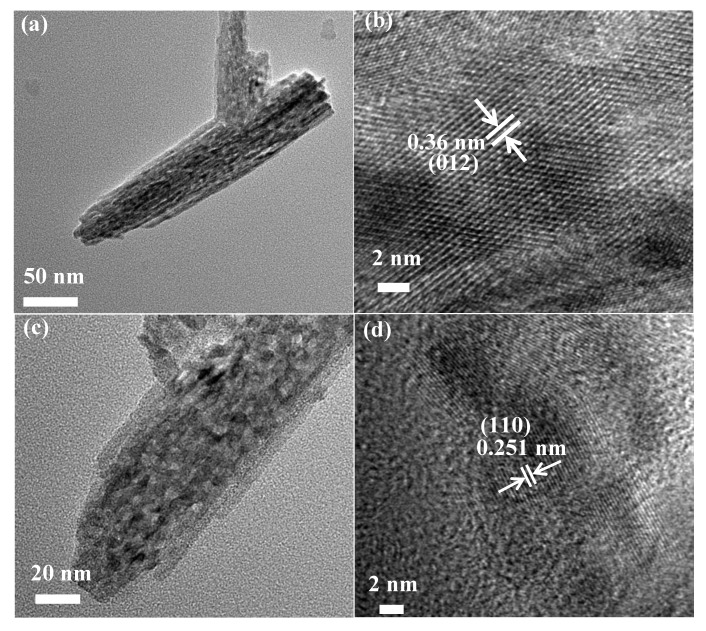
(**a**) TEM image of α-Fe_2_O_3_ nanowire arrays (**b**) HRTEM image of α-Fe_2_O_3_ nanowire arrays (**c**) TEM image of α-Fe_2_O_3_@C nanowire (**d**) HRTEM image of α-Fe_2_O_3_@C nanowire.

In order to explore the microstructure of the material, the TEM image of α-Fe_2_O_3_ nanowire arrays is shown in Figure 2a. Further information about the microstructure and phase of the single α-Fe_2_O_3_ nanowire arrays was studied by a high-resolution transmission electron microscopy (HRTEM) image shown in Figure 2b, in which the measured interplanar spacings of 0.36 nm for well-defined lattice fringes consist of the α-Fe_2_O_3_ (012) planes. The TEM image of α-Fe_2_O_3_@C nanowire arrays is shown in Figure 2c. As can be seen from the image, the material presents a porous structure, with a 5 nm carbon shell. HRTEM image is shown in Figure 2d, in which the measured interplanar spacings of 0.25 nm for well-defined lattice fringes consist of the α-Fe_2_O_3_ (110) planes.

**Figure 3:**
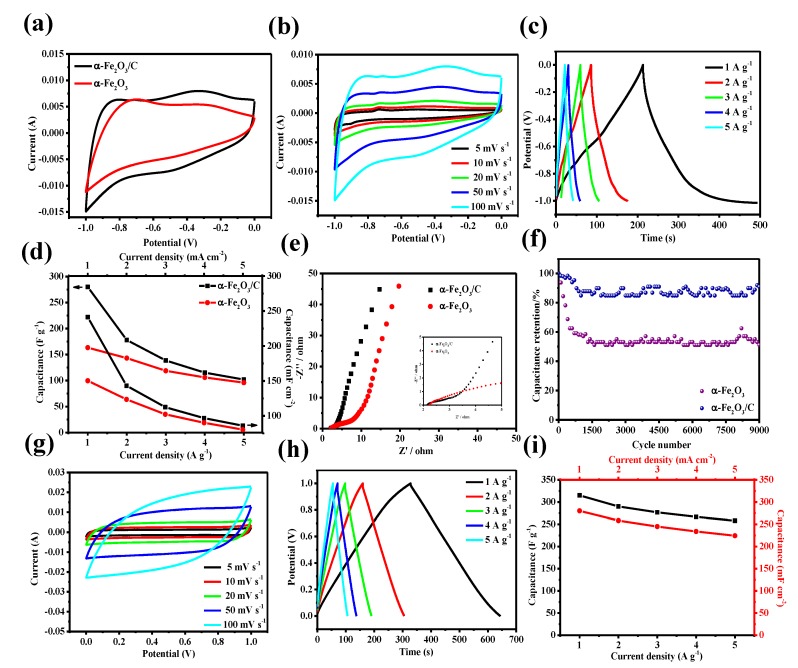
(**a**) CV curves of α-Fe_2_O_3_ nanowire arrays and α-Fe_2_O_3_@C nanowire electrode at a scan rate of 100 mV s^−1^ (**b**) CV curves of α-Fe_2_O_3_ nanowire arrays electrode at different scan rates (**c**) GCD curves of α-Fe_2_O_3_@C nanowire electrode at varied current density (**d**) The specific mass capacitance and area capacitance of α-Fe_2_O_3_ nanowire arrays and α-Fe_2_O_3_@C nanowire electrodes calculated from CGD curves (**e**) Nyquist plots for α-Fe_2_O_3_ nanowire arrays andα-Fe_2_O_3_@C nanowire electrodes (**f**) Cycle performance of α-Fe_2_O_3_ nanowire arrays andα-Fe_2_O_3_@C nanowire electrode at 5 A g^−1^ for 9000 cycles (**g**) CV curves of MnO_2_ nanosheets electrode at different scan rates (**h**) GCD curves of MnO_2_ nanosheets electrode at varied current densities (**i**) The specific mass capacitance and area capacitance of MnO_2_ nanosheets electrode calculated from CGD curves.

The electrochemical performances of α-Fe_2_O_3_@C nanowire arrays electrode are measured in a three-electrolyte cell with 1 M Na_2_SO_4_ as the electrolyte in a potential window from −1 to 0 V (vs. Ag/AgCl). In Figure 3a, the cyclic voltammetry (CV) curve of the α-Fe_2_O_3_@C nanowire arrays electrode exhibits larger capacitive current density than a single α-Fe_2_O_3_ nanowire arrays electrode. According to the CV curve shapes, it can be found that the capacitance of the α-Fe_2_O_3_@C nanowire arrays electrode can be attributed to the electrical double layer capacitor (EDLC) by the surface adsorption of electrolyte ions and the pseudo capacitance of α-Fe_2_O_3_ by the redox couple of Fe^2+^/Fe^3+^ [32]. The CV curve of the α-Fe_2_O_3_@C nanowire arrays electrode is expanded, suggesting that the hybrid core-shell electrode can improve the capacitance of the α-Fe_2_O_3_ nanostructures. Figure 3b shows the CV curves of the α-Fe_2_O_3_@C nanowire arrays electrode at various scan rates from 5 to 100 mV s^−1^. With the increase of the scan rate, the CV curves of the α-Fe_2_O_3_@C nanowire arrays electrode can still retain a definite rectangular shape. Figure 3c is the GCD curves of the α-Fe_2_O_3_@C nanowire arrays electrode at different current densities from 1 to 5 A g^−1^. The mass specific capacitance and area specific capacitance of the two electrodes are calculated from GCD curves, (Figure 3d). It can be determined that the α-Fe_2_O_3_@C nanowire arrays electrode delivers a higher specific capacitance of 280 F g^−1^ and 241.3 mF cm^−2^ at a current density of 1 A g^−1^ and 1 mA cm^−2^, respectively. Whereas only specific capacitances of 163.3 F g^−1^ and 150.3 mF cm^−2^ are obtained for the single α-Fe_2_O_3_ nanowire arrays electrode. EIS of the α-Fe_2_O_3_@C nanowire arrays electrode and the single α-Fe_2_O_3_ nanowire arrays electrode are carried out. All EIS spectra show two distinct parts consisting of a semicircle in the high frequency region (charge transfer process) and a sloped straight line in the low frequency region (diffusion-limited process). As shown in Figure 3e, the α-Fe_2_O_3_@C nanowire arrays electrode presents a smaller equivalent series resistance (R_s_) of 2.15 Ω and the resistance of the α-Fe_2_O_3_ nanowire arrays electrode is 2.24 Ω. The carbon shell can increase the conductivity of the electrode and accelerate the electron transfer rate. The corresponding cycling stabilities of the α-Fe_2_O_3_@C nanowire arrays electrode and the α-Fe_2_O_3_ nanowire arrays electrode are evaluated by GCD at a scan rate of 5 A g^−1^ (Figure 3f), demonstrating a higher capacitance retention of 90% after 9000 cycles than the single α-Fe_2_O_3_ nanowire arrays electrode (51% retention after 9000 cycles).

Similarly, the electrochemical performance of MnO_2_ nanosheets electrode is also measured in a three-electrolyte cell with 1 M Na_2_SO_4_ as the electrolyte in a potential window from 0 to 1 V (vs. Ag/AgCl). The CV curves of MnO_2_ nanosheets electrode are measured at a different scan rate from 5 to 100 mV s^−1^, the quasi-rectangular shape of the CV curves is well preserved, revealing a good rate capability of the electrode material (Figure 3g) [33]. The GCD curves of MnO_2_ nanosheets electrode at different current densities from 1 to 5 A g^−1^ are shown in Figure 3h. The specific capacitance of MnO_2_ nanosheets electrode is calculated from GCD curves (Figure 3i). And the highest specific capacitance is 315 F g^−1^ at a current density of 1 A g^−1^.

**Figure 4:**
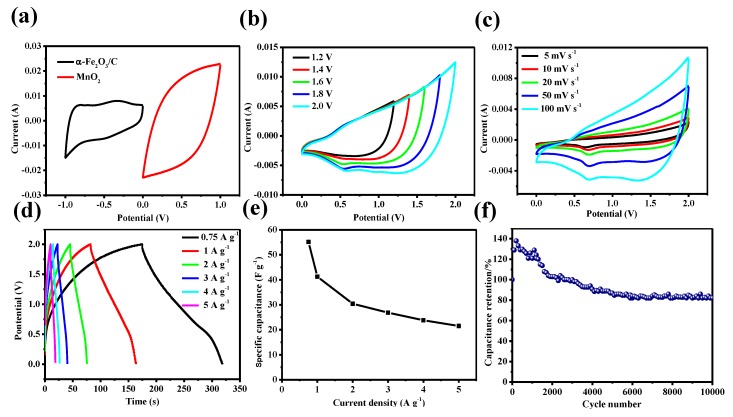
(**a**) CV curves of α-Fe_2_O_3_@C nanowire electrode and MnO_2_ nanosheets electrode at a scan rate of 100 mV s^−1^ (**b**) CV curves of ASC in different potential windows (**c**) CV curves of ASC at different scan rates in a voltage range between 0 and 2 V (**d**) GCD curves of ASC device at different current densities (**e**) The specific capacitance of the ASC device calculated from GCD curves (**f**) Cycle performance of ASC.

Figure 4a shows CV curves of the α-Fe_2_O_3_@C nanowire arrays electrode and MnO_2_ nanosheets electrode in separate potential windows of −1–0 V and 0–1 V at a scan rate of 100 mV s^−1^. Based on the separate potential windows and matchable capacitance characteristics, an ASC based α-Fe_2_O_3_@C nanowire arrays anode and MnO_2_ nanosheets cathode could achieve 2 V cell voltage theoretically. Figure 4b shows CV curves of the as-assembled α-Fe_2_O_3_@C//MnO_2_ ASC device with an operation voltage window ranging from 0 to 2 V at the scan rate of 100 mV s^−1^, revealing the α-Fe_2_O_3_@C//MnO_2_ ASC is stable up to an operational voltage of 2 V. Figure 4c is CV curves of the α-Fe_2_O_3_@C//MnO_2_ ASC. At different scan rates, the shape of the CV curves exhibit the triangle characteristic, which show good stability. The GCD curves at different current densities are showed in Figure 4d, and the shapes of all curves are triangular, further confirming excellent capacitive performance of the α-Fe_2_O_3_@C//MnO_2_ ASC. As shown in Figure 4e, the specific capacitance of the α-Fe_2_O_3_@C//MnO_2_ ASC is calculated from GCD curve, and a maximum capacitance of 55.125 F g^−1^ at a discharge current density of 0.75 A g^−1^. The cyclic durability of the α-Fe_2_O_3_@C//MnO_2_ ASC is further evaluated at 4 A g^−1^ for 10,000 cycles and it can be seen that the ASCs retains 82% of the initial capacitance, proving its good cycling performance (Figure 4f).

**Figure 5:**
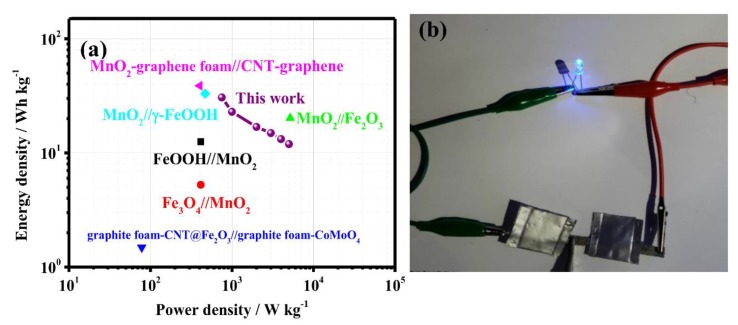
(**a**) Ragone plots of ASC (**b**) Digital image of blue light-emitting diodes (LED) lighted by the α-Fe_2_O_3_@C//MnO_2_ ASC device.

Figure 5a shows the Ragone plots of the α-Fe_2_O_3_@C//MnO_2_ ASC. It is significant that the as-assembled ASC device can achieve an energy density of 30.625 Wh kg^−1^ at a current density of 0.75 A g^−1^, and an energy density of 11.944 at 5 A g^−1^. Such an energy density is superior to those of the recently reported ASC device, such as FeOOH//MnO_2_ (12 Wh kg^−1^) [34], Fe_3_O_4_//MnO_2_ (8 Wh kg^−1^) [35], MnO_2_//Fe_2_O_3_ (19.4 Wh kg^−1^) [36], graphite foam-CNT@Fe_2_O_3_//graphite foam-CoMoO_4_ (1.4 Wh kg^−1^) [37], MnO_2_-graphene foam//CNT-graphene (31.8 Wh kg^−1^) [38], MnO_2_//γ-FeOOH (37.4 Wh kg^−1^) [39]. To show the practical application of the α-Fe_2_O_3_@C//MnO_2_ ASC, the prototype device is connected to a 3.2 V blue light emitting diode (LED) and can successfully lighten it for 3 min (Figure 5b).

## 4. Conclusions

In summary, the α-Fe_2_O_3_@C nanowire arrays electrodes have been prepared through a facile hydrothermal route. The hybrid electrode material can yield an enhanced mass capacitance of 280 F g^−1^ at 1 A g^−1^, which can be attributed to a high utilization of carbon shell and a fast charge transport in the electrode. Moreover, the α-Fe_2_O_3_@C nanowire arrays electrode exhibits an excellent cyclic stability with 90% retention after 9000 cycles. With well-separated potential windows and matchable specific capacitances, a flexible high-performance ASC device with 2.0 V voltage has been constructed based on the α-Fe_2_O_3_@C nanowire arrays electrode as the anode and MnO_2_nanosheets electrode as the cathode, demonstrating a maximum energy density of 30.625 Wh kg^−1^ and a good rate capability. Moreover, this facile methodology enables the development of new functional hybrid systems with metal current collectors and nanostructured active materials, which could find various applications in electrochemical devices in the future.
